# Spectroscopic Microtomography in the Short-Wave Infrared Wavelength Range

**DOI:** 10.3390/s23115164

**Published:** 2023-05-29

**Authors:** Cory Juntunen, Andrew R. Abramczyk, Peter Shea, Yongjin Sung

**Affiliations:** College of Engineering and Applied Science, University of Wisconsin-Milwaukee, 3200 N Cramer St., Milwaukee, WI 53211, USA

**Keywords:** near-infrared imaging, optical tomography, spectroscopy

## Abstract

Spectroscopic microtomography provides the ability to perform 4D (3D structural and 1D chemical) imaging of a thick microscopic specimen. Here, we demonstrate spectroscopic microtomography in the short-wave infrared (SWIR) wavelength using digital holographic tomography, which captures both the absorption coefficient and refractive index. A broadband laser in tandem with a tunable optical filter allows us to scan the wavelength from 1100 to 1650 nm. Using the developed system, we measure human hair and sea urchin embryo samples. The resolution estimated with gold nanoparticles is 1.51 μm (transverse) and 1.57 μm (axial) for the field of view of 307 × 246 μm${}^{2}$. The developed technique will enable accurate and efficient analyses of microscopic specimens that have a distinctive absorption or refractive index contrast in the SWIR range.

## 1. Introduction

Spectroscopic microtomography records the absorption spectrum (i.e., the absorption coefficient as a function of the wavelength of the interrogating light) at every voxel in the three-dimensional (3D) volume of a microscopic specimen. It has been demonstrated in the visible [1,2], mid-infrared (MIR) [3,4], and soft X-ray ranges [5,6], showcasing the value of the information contained within the 4D (3D volume and 1D spectrum) data cube of a wide range of samples. In this paper, we demonstrate spectroscopic microtomography in the short-wave infrared (SWIR) range of 1100–1650 nm, which is the near-infrared (NIR) range where InGaAs cameras produce high sensitivity. Vibrational spectroscopy using infrared absorption or Raman scattering has long been explored as a non-invasive chemical analysis tool as it can provide the molecular fingerprints of a variety of materials and chemical compounds [7]. In comparison with MIR [8] or Raman [9] spectroscopy, NIR spectroscopy in the wavelength range of 800–2500 nm offers different opportunities for chemical imaging [10]. Using overtones and combination bands, the NIR spectrum is not as specific as the MIR or Raman spectra; however, NIR light penetrates more deeply, and therefore can be used to image thicker specimens. Many materials of practical interest have a distinct NIR spectrum, which, in combination with a proper chemometric analysis, allows for accurate classification and regression at much lower time and cost than MIR or Raman spectroscopy. Microscopic imaging using NIR can provide a high resolution with minimal sample preparation and without concern about sample heating [10]. In addition, using NIR, thick biological samples can be imaged in the culture medium in their most native condition, whereas MIR would require compression or dehydration of thick samples.

There are several strategies for spectroscopic microtomography which allow for the 3D spatial reconstruction of a sample while also providing the spectrum at each voxel in the 3D space, resulting in a 4D data cube. Our approach utilizes digital holographic tomography (DHT) and a wavelength-scanning light source via a broadband white-light laser and tunable filter, as well as a variable optical path length (VOPL) strategy. DHT can provide the 3D maps of absorption and refractive index by recording both the amplitude and phase images for varying orientations of the sample with respect to the illumination beam. The relative orientation of the sample can be varied by rotating the sample around an axis orthogonal to the optical axis [11], rotating the illumination beam [12,13], or both [14,15]. We use beam-rotation tomography, which is much faster than sample-rotation tomography but suffers more from the missing-cone artefact due to the insufficient angular coverage of the incident beam. DHT is also called tomographic phase microscopy [13], tomographic diffractive microscopy [16], etc. We use a continuum laser with a tunable bandpass filter, which allows for spectroscopic interrogation at selected wavelengths bypassing the lengthy interferogram collection process of Fourier transform spectroscopy (FTS). FTS can provide a high spectral resolution, but it requires the collection of the entire interferogram, which typically consists of more than 1000 samples. Using DHT in combination with a wavelength-scanning laser, we previously demonstrated spectroscopic microtomography in the visible wavelength range, measuring the absorption spectra of oxygenated and deoxygenated blood cells [1]. In this work, we have constructed a new system for spectroscopic microtomography in the SWIR range. Using the developed system, we measure the refractive index and absorption spectrum of microscopic specimens for the wavelength range of 1100 to 1650 nm. Although the maximum numerical aperture of the NIR objective lenses was only 0.65, allowing us to change the illumination angle by up to 40°, we were still able to perform 3D imaging of a human hair and sea urchin embryos. Using gold nanoparticles, we evaluated the 3D imaging performance of the developed system. The demonstrated method opens a new door to chemical imaging of complex microscopic specimens that show absorption or refractive index contrast in the SWIR range.

## 2. Materials and Methods

### 2.1. System Design

**Figure 1:**
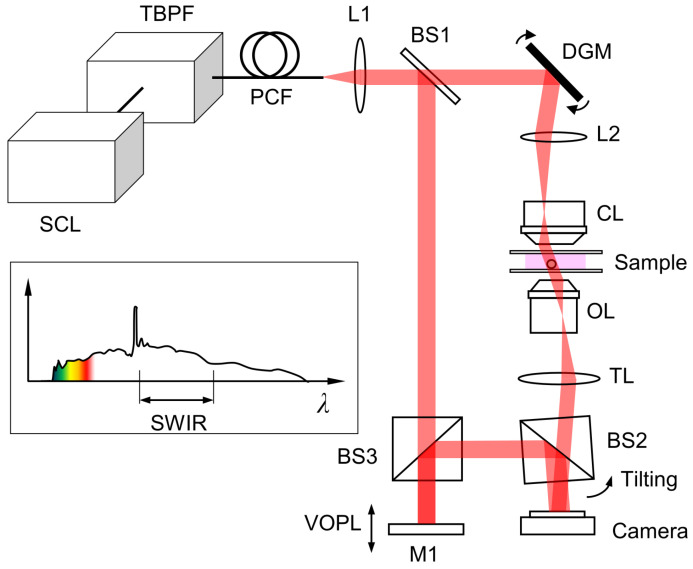
Schematic diagram of the 4D NIR spectral-microtomographic system. As the source light enters the optical system, the sample and reference beam paths are split and combined before images are acquired. SCL: supercontinuum laser; TBPF: tunable bandpass filter; PCF: photonic crystal fiber; L1 and L2: lenses; M1: mirror; BS1, BS2 and BS3: beam splitters; DGM: dual-axis galvanometer mirror; CL: condenser lens; OL: objective lens; TL: tube lens; VOPL: variable optical path length.

Our 4D SWIR system was built in the lab starting with the supercontinuum laser light source (SCL) (NKT Photonics, WL SC400-4) which generates broadband white-light. This white light passes through a tunable bandpass filter (TBPF) (NKT Photonics, SWIR HP8), allowing a spectral band of light narrower than 5 nm (FWHM) to pass through, which is controlled by LabVIEW code. A SuperK Connect broadband fibre-coupling unit couples the light to a photonic crystal fibre (PCF), which connects to a cage mount on our optical system. After the PCF, the beam passes through an achromat (L1) with a focal length of 100 mm, and an iris for alignment purposes. The beam passes through a beam splitter (BS1) which splits the beam into a sample and a reference beam path. The sample beam rotates with dual galvanometer mirrors (DGM) (Cambridge Technology, 8310KM60) to change the angle of incidence of the beam by up to 40° with respect to the optical axis. These galvanometer mirrors are controlled by an NI controller and LabVIEW code. After the galvanometer mirrors, the beam passes through the condenser lens (CL) (Mitutoyo, 50×, 0.65 NA) and through the sample which is placed in an appropriate index matching oil and sandwiched between two cover glasses. Next, the beam passes through the objective lens (OL) (Mitutoyo, 50×, 0.65 NA) and tube lens (TL) with a focal length of 100 mm. Two additional lenses with focal lengths of 75 mm and 125 mm (not shown in Figure 1) are installed before combining with the reference beam. All the lenses in the sample beam path are installed in the 4-f telecentric configuration. For the reference beam, the beam passes through a beam splitter (BS3) and reflects off of a mirror (M1). Mirror M1 is placed on a motorized stage (Thorlabs, MTS50-Z8) which is used to adjust the variable optical path length (VOPL) of the reference beam. Finally, the reference beam passes through another beam splitter (BS2) where the reference beam is combined with the sample beam, producing interference fringes. BS2 is rotated slightly so that the interference between the sample and reference beams produces vertical fringes as opposed to bullseye pattern fringes. An InGaAs camera (Raptor Photonics, OWL 1.7-VS-CL-1280) composed of 1280 × 1024 pixels of 10 μm is used to record the raw interferogram images. The overall magnification factor is 41.7, corresponding to the field of view of 307 × 246 μm${}^{2}$, and the pixel resolution is 0.24 μm. The off-axis DHT reduces the resolution by a factor of 3, and the Nyquist criterion further reduces the effective resolution by another factor of 2. Thus, the theoretical resolution of the current design is about 1.44 μm. All optical elements used within the system utilize an anti-reflective “C” coating which is optimized for the wavelengths scanned in this demonstration. An additional white-light LED source and a charge-coupled device (CCD) camera (Allied Vision, Pike) are installed to acquire a bright-field image of the same sample. The DHT and bright-field imaging modes are switched using a flip mirror installed between DGM and L2. A program allowing control of all the mentioned equipment is implemented in LabVIEW (National Instruments).

### 2.2. Sample Preparation

A crown hair sample was collected from a Caucasian male. To prepare the hair sample for imaging, we first adhered it to a 1 mm thick microscope slide using double-sided tape. The slide was then placed under a bright-field microscope. Using a flat-bladed Exacto knife in our left hand, we held the hair sample steady at an angle almost parallel to the surface of the microscope slide. We positioned a light source above our left hand, angled towards the right, to allow light to pass through the sample into the microscope. With another flat-bladed Exacto knife held vertically in our right hand, perpendicular to the microscope slide, we made a downward-chopping motion to slice the hair sample into lengths of 150–200 μm. The hair sample was placed in index matching oil with a refractive index of 1.55 (Cargille Labs) to minimize the diffraction effect. Polystyrene microspheres (Polysciences, Inc.) of 20 μm in diameter were immersed in the immersion oil (Cargille Labs), with a refractive index 1.57, sandwiched between two round coverslips (No. 1, 35 mm diameter), and imaged to determine the iteration number to alleviate the missing cone problem. Custom-made sea urchin embryo slides were purchased from Carolina Biological Supply Company. The undyed embryos in different developmental stages were fixed and mounted with Eukitt mounting medium (Electron Microscopy Sciences, 15,320) between two round coverslips. Gold nanoparticles of 100 nm in diameter were purchased from nanoComposix, Inc. To resuspend the settled nanoparticles, the bottle containing the colloid was vigorously shaken for about 30 s. A 10 μL liquid drop of suspension was evenly spread onto a No. 1 round coverslip of 35 mm in diameter and dried in air. A drop of Eukitt was added onto the coverslip and covered with another round coverslip. The sample was imaged after about 2 h. The refractive index of the Eukitt in the SWIR range was measured using synthetic phase microscopy, and given as $n\left(\lambda \right)=1.4736+1.4887\times 10^{-4}\lambda^{2}+0.0068/\lambda^{2}$, where λ is the wavelength in μm.

### 2.3. Data Acquisition

Using bright-field imaging, the sample regions were easily located. Once a sample region was found, the LED light source was turned off, and the flip mirror was moved out of the beam path, allowing the SWIR light to pass from the continuum laser and tunable filter through the galvanometer mirrors and sample, through the rest of the system. The DHT data were acquired in the 1100–1650 nm wavelength range at a 50 nm step size. For each wavelength, 400 projection images were acquired. The starting position of the mirror M1 on the motorized stage was adjusted so that the fringes in the field of view had strong and equal contrast at 1350 nm. Due to the effect of chromatic aberration, each wavelength had an optimal mirror position giving the highest contrast. The optimal mirror positions were manually determined in a calibration experiment, where the mirror was translated along the optical axis until the image with highest interference fringe contrast was found for each wavelength. These mirror locations were recorded and then used by the LabVIEW program to automatically translate the mirror so that the interference fringes remained at the highest contrast as the wavelength was scanned. At each wavelength, the galvanometer scanner changed the angle of incidence, allowing us to capture 400 images of the sample at varying angles of up to 40°. After acquiring the sample dataset, another set of images was acquired after moving the sample out of the field of view. The background dataset was acquired only once and used for all the sample datasets. The total data acquisition time to collect 12 wavelength stacks of 3D tomograms (400 images per each tomogram) was about 7 min for each sample and background dataset.

### 2.4. Data Processing

For each wavelength λ, a series of interferogram images was recorded while changing the illumination direction, $\vec{k_{0}}$. From each raw interferogram image, the amplitude and phase distributions of the light was extracted using a standard fringe analysis technique [17]. Using Equations (1a) and (1b), we normalized the amplitude image for the sample againsr the background (i.e., the amplitude image recorded without the sample) and subtracted the background phase image from the sample.

(1a) $$\begin{array}{cc} A(x,y;\lambda ,\vec{k_{0}}) & =A^{\left(s\right)}\left(x,y;\lambda ,\vec{k_{0}}\right)/A^{\left(b\right)}\left(x,y;\lambda ,\vec{k_{0}}\right), \end{array}$$

(1b) $$\begin{array}{c} \begin{array}{cc} \Phi (x,y;\lambda ,\vec{k_{0}}) & =\Phi^{\left(s\right)}\left(x,y;\lambda ,\vec{k_{0}}\right)-\Phi^{\left(b\right)}\left(x,y;\lambda ,\vec{k_{0}}\right). \end{array} \end{array}$$

**Figure 2:**
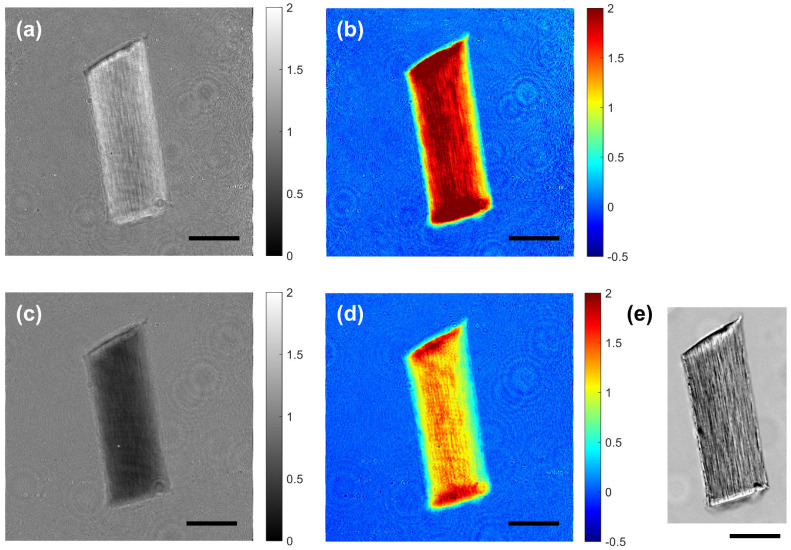
Example images acquired with the developed system. (**a**,**b**) The amplitude and phase images of a hair, respectively, imaged at 1100 nm. (**c**,**d**) The amplitude and phase images of the same hair, respectively, imaged at 1500 nm. The amplitude images in (**a**,**c**) are normalized with the background images, i.e., the amplitude images acquired without the sample at the corresponding wavelengths. The phase images in (**b**,**d**) are shown after subtracting the background images, i.e., the phase images acquired without the sample at the corresponding wavelengths (unit: radian). The amplitude and phase images for the two different wavelengths (1100 and 1500 nm) clearly show the wavelength-dependent absorption and refractive index properties of the hair. (**e**) A bright-field image of the same hair. Scale bars: 50 μm.

Figure 2 shows examples of the amplitude and phase images acquired with the system shown in Figure 1. Figure 2a and Figure 2b show the amplitude and phase images acquired at 1100 nm, respectively. Figure 2c and Figure 2d show the amplitude and phase images acquired at 1500 nm, respectively. Figure 2e is a bright-field image of the same sample.

For each wavelength and illumination direction, a scattered light field, $U_{s}\left(x,y;\lambda ,\vec{k_{0}}\right)$, can be synthesized. With the first-order Born approximation [18], the scattered light field was synthesized using

(2) $$U_{s}\left(x,y;\lambda ,\vec{k_{0}}\right)=A\left(x,y;\lambda ,\vec{k_{0}}\right)\exp\left[i\Phi (x,y;\lambda ,\vec{k_{0}})\right]-1.$$

With the first-order Rytov approximation [19], the scattered light field was synthesized using

(3) $$U_{s}\left(x,y;\lambda ,\vec{k_{0}}\right)=\ln\left(A\left(x,y;\lambda ,\vec{k_{0}}\right)\right)+i\Phi \left(x,y;\lambda ,\vec{k_{0}}\right).$$

The scattering potential F(X,Y,Z) can be defined for an imaged sample as a function of the absorption and refractive index properties.

(4) $$F\left(X,Y,Z\right)=-{\left(2\pi /\lambda \right)}^{2}\left({\left[n(X,Y,Z)+ik(X,Y,Z)\right]}^{2}-n_{m}^{2}\right),$$

where $n_{m}$ is the refractive index of the medium in which the sample is immersed, n(X,Y,Z) is the 3D refractive index distribution in the sample, and k(X,Y,Z) is the 3D distribution of absorption constant, which can be related to the absorption coefficient μ(X,Y,Z) by μ(X,Y,Z)=(4π/λ)k(X,Y,Z).

**Figure 3:**
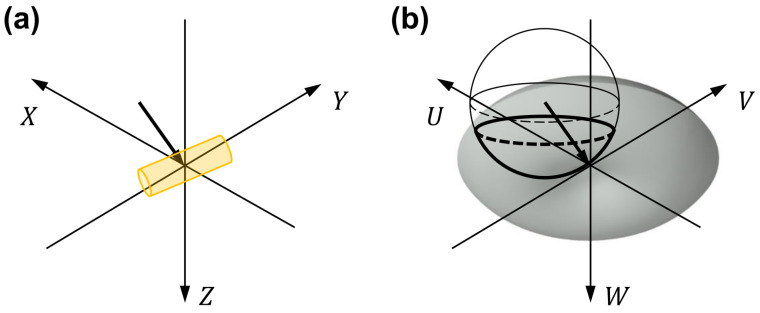
Digital holographic tomography data processing. For each wavelength, the amplitude and phase images recorded for an illumination angle are used to synthesize a scattered light field from the sample. The arrow in (**a**) represents the wave vector $\vec{k_{0}}=\left(u_{o},v_{0},w_{0}\right)$ of the illumination beam, whose magnitude and direction correspond to $n_{m}/\lambda$ and the illumination direction onto the sample plane, respectively. As shown in (**b**), the Fourier transform of the scattered light field is mapped onto the Ewald’s sphere, which is shifted in the opposite direction to the wave vector, in the 3D spatial frequency space. (X,Y,Z) are the Cartesian coordinates with *Z* being the optical axis direction. (U,V,W) are the spatial frequency components corresponding to (*X*,*Y*,*Z*), respectively.

The scattered light fields given in Equations (3) and (4) can be related to the scattering potential of the imaged specimen, V(x,y,z), in Equation (5) [20]. The first-order Born approximation is valid when the size of the imaged object is very small, whereas the first-order Rytov approximation is valid when the refractive index increment is small at the boundaries. For hair samples, the first-order Rytov approximation is more appropriate, because the hair is thick, but its absorption constant *k* is only about $5\times 10^{-3}$, corresponding to the absorption coefficient μ of 400 cm${}^{-1}$ at 1500 nm. The measured absorption coefficient of the sea urchin embryos is also small; thus, the use of the first-order Rytov approximation is justified. For gold nanoparticles, the refractive index of gold is very different from that of water or mounting medium used. Thus, using the first-order Born approximation is more appropriate.

(5) $$\tilde{U_{s}}\left(U,V;\lambda ,\vec{k_{0}}\right)=\frac{\pi }{iw}\tilde{F}\left(U-u_{0},V-v_{0},W-w_{0}\right),$$

where $\tilde{U_{s}}$ and $\tilde{F}$ are the 2D Fourier transform of $U_{S}$ and the 3D Fourier transform of *F*, respectively, $W=\sqrt{{\left(n_{m}/\lambda \right)}^{2}-U^{2}-V^{2}}$, and $\left(u_{0},v_{0},w_{0}\right)$ are the components of the wave vector $\vec{k_{0}}$ for the incident beam, which satisfy $u_{0}^{2}+v_{0}^{2}+w_{0}^{2}={\left(n_{m}/\lambda \right)}^{2}$. A graphical interpretation of Equation (4) maps the Fourier transform of the scattered field given in Equations (2) or (3) to the spherical surface called the Ewald’s sphere in the 3D spatial frequency space, as shown in Figure 3.

The Ewald’s sphere, onto which the scattered field is mapped, is shifted along different directions as the illumination angle is varied [18]. Thus, from the scattered fields measured for varying illumination angles, the 3D object spectrum (i.e., the components of $\tilde{F}\left(U,V,W\right)$) can be retrieved for many (U,V,W) values. After completing the mapping, we take the 3D inverse Fourier transform, which provides the scattering potential of the sample, and thus the 3D maps of the refractive index and absorption constant (or absorption coefficient). Of note, the refractive index of the reconstructed tomogram is underestimated due to the incomplete angular sampling, which is known as the missing cone artefact [21]. The absorption coefficient can also be underestimated and even negative due to the missing cone. For polystyrene microspheres, 300 iterations produced a mean refractive index value equal to that measured with a different method. The same iteration number is used for the reconstruction of the hair, which also has a circular cross section.

## 3. Results

### 3.1. 3D Maps of the Absorption and Refractive Index of Human Hair

**Figure 4:**
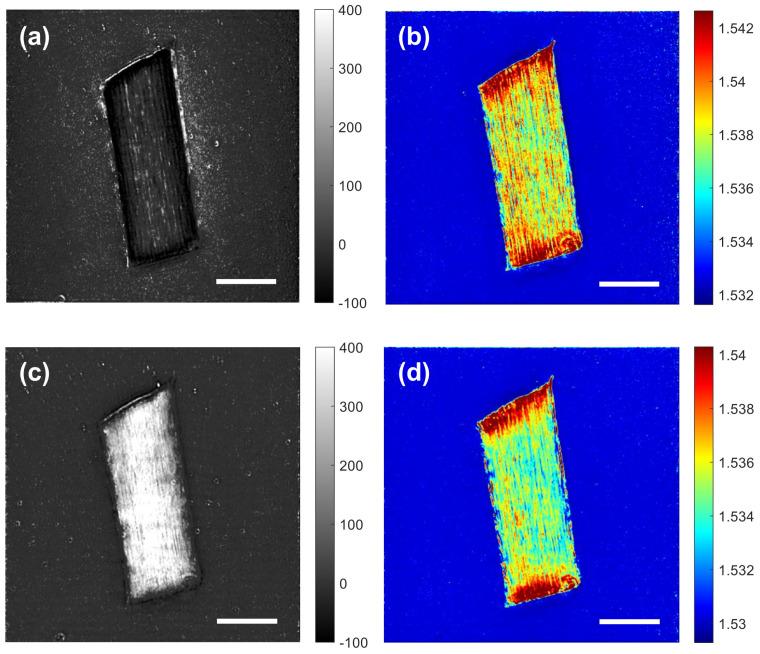
Examples of the absorption coefficient and refractive index maps reconstructed from a series of scattered field measurements. (**a**,**b**) The absorption coefficient and refractive index maps, respectively, for the centre cross-section at a wavelength of 1100 nm. (**c**,**d**) The absorption coefficient and refractive index maps, respectively, for the centre cross-section at a wavelength of 1500 nm. The unit for the absorption coefficient is cm${}^{-1}$. Scale bars: 50 μm.

Figure 4 shows examples of the absorption coefficient and refractive index maps obtained from the DHT measurement. At a wavelength of 1100 nm, the absorption coefficient of the hair is very small, as shown in Figure 4a. The negative absorption coefficient is an artefact due to the missing cone, the coherent speckle, or both. Figure 4b shows that the refractive index of the hair is about 1.538 at a wavelength of 1100 nm. The high refractive index near the edges is an artefact, which is excluded in calculating the mean refractive index. As shown in Figure 4c, the absorption coefficient dramatically increases to about 400 cm${}^{-1}$ as the wavelength is increased to 1500 nm, while the refractive index decreases to about 1.534.

**Figure 5:**
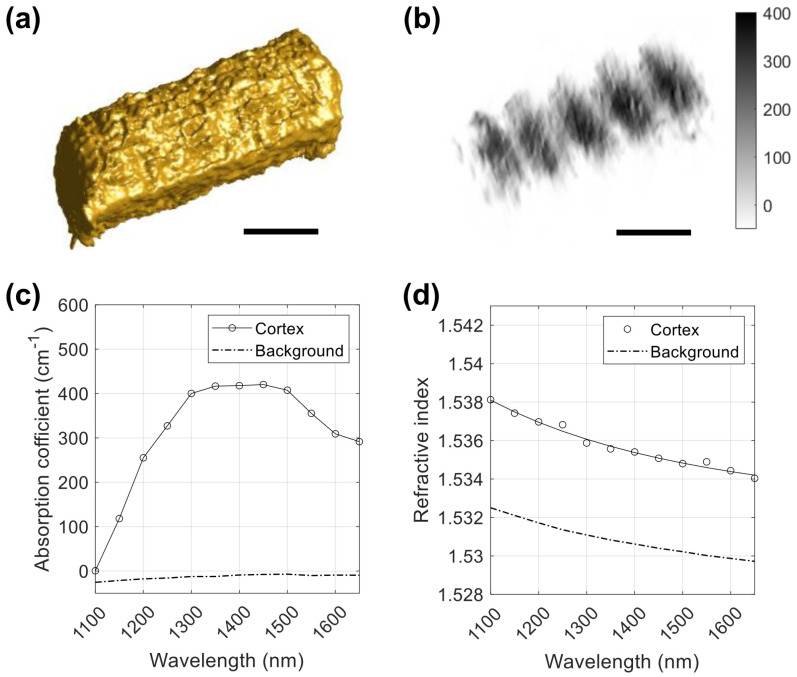
(**a**) a 3D-rendered image of hair calculated from the 3D refractive index map at a wavelength of 1100 nm. Although the optical system is capable of a 5 nm spectral resolution, the step size of 50 nm was found to provide adequate spectral resolution to capture the relevant characteristics of the absorbance of the hair sample in the SWIR wavelength range. (**b**) An example of the 3D absorption coefficient map of hair at a wavelength of 1500 nm. Five vertical cross-sections at 30 μm intervals are shown as an example. (**c**,**d**) The mean absorption coefficient and mean refractive index value, respectively, of the hair sample as a function of the wavelength in the 1100–1650 nm range. Scale bars in (**a**,**b**): 50 μm.

Figure 5a shows a 3D-rendered image of the hair, calculated from the 3D refractive index map obtained at 1100 nm. Figure 5b shows vertical cross-sections of the hair at 30 μm intervals, extracted from the 3D absorption coefficient map measured at a wavelength of 1500 nm. In the 3D absorption map at 1500 nm, we expect to see a combination of water bands and weak protein bands. Figure 5c and Figure 5d show the mean absorption coefficient and mean refractive index value, respectively, in a wavelength range of 1100–1650 nm. The shape of the measured absorption coefficient matches with the absorbance profiles obtained with diffuse reflectance measurements [22,23,24]. We could not find the refractive index dispersion of hair in the literature. The refractive index of the hair shown in Figure 5d can be fitted to the Cauchy–Schott equation $n\left(\lambda \right)=1.5305+1.3649\times 10^{-4}\lambda^{2}+0.0089/\lambda^{2}$ with an RMSE of $2.0\times 10^{-4}$. To the best of our knowledge, this is the first report of the absorption coefficient and refractive index of human hair in the SWIR range. We note that human hairs strongly absorb visible light; thus, the absorption coefficient of a human hair cannot be obtained using the visible-light DHT system, which was presented in our previous publication [1].

### 3.2. 3D Refractive Index Imaging of Sea Urchin Embryos in Different Developmental Stages

**Figure 6:**
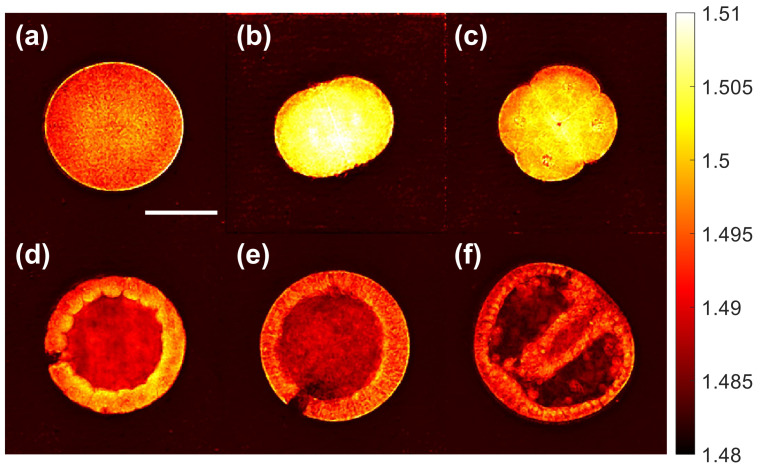
Refractive index cross-sections of the sea urchin embryos in different developmental stages: (**a**) egg, (**b**) two cells, (**c**) four cells, (**d**) morula, (**e**) blastula, (**f**) gastrula. The refractive index was measured at a wavelength of 1100 nm. The scale bar (50 μm) in (**a**) applies to all the images.

**Figure 7:**
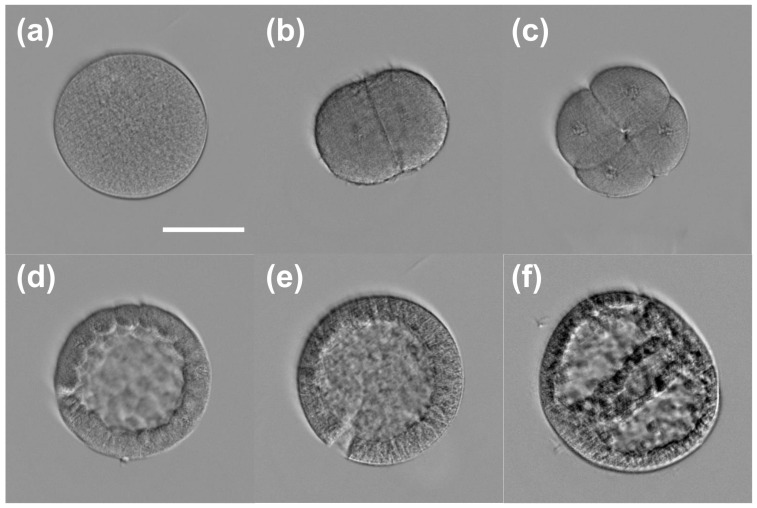
Bright-field images of the sea urchin embryos in different developmental stages: (**a**) egg, (**b**) two cells, (**c**) four cells, (**d**) morula, (**e**) blastula, (**f**) gastrula. The scale bar (50 μm) in (**a**) applies to all the images.

**Figure 8:**
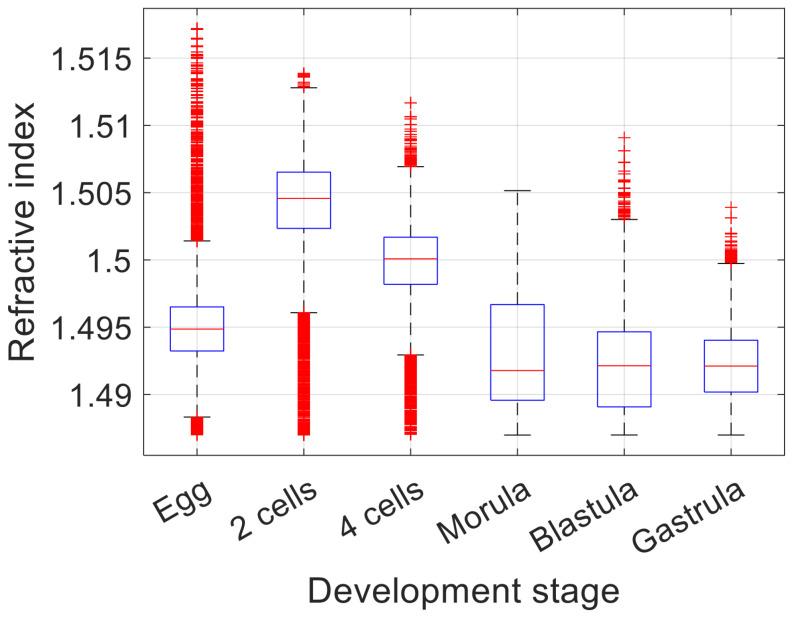
Refractive index of the sea urchin embryos in different developmental stages measured at a wavelength of 1100 nm. In each box, the central mark (red) indicates the median, the bottom and top edges (blue) indicate the 25th and 75th percentiles, respectively, and the whiskers (black) mark the range of data points not considered outliers (red crosses).

We performed 3D refractive index imaging of sea urchin embryos in different developmental stages: egg, two cells, four cells, morula, blastula, and gastrula. Figure 6 shows the horizontal cross-section of each embryo at a wavelength of 1100 nm. For comparison, we acquired bright-field images of the same samples, shown in Figure 7. In Figure 6, we can see that the embryos in the two-cell and four-cell stages have higher refractive index values than the other embryos in the egg, morula, blastula, and gastrula stages. The two-cell and four-cell embryos also smaller in size. Considering that the refractive index is proportional to the density of non-aqueous content (i.e., dry mass) in a biological cell [25,26], Figure 6 and Figure 7 show that sea urchin embryos experience a change in the mass density through volume contraction and expansion. For a more quantitative analysis, we selected the intracellular region, whose refractive index value is higher than that of the background medium by at least 0.005. Figure 8 shows the distribution of the refractive index values in the selected region for each embryo. In each box, the central mark indicates the median, the bottom and top edges indicate the 25th and 75th percentiles, respectively, and the whiskers mark the range of data points not considered outliers. Figure 8 shows that the median refractive index of the two-cell embryo was 1.505, 0.010 higher than the egg (1.495). The median refractive index of the four-cell embryo was 1.500, 0.005 lower than the two-cell embryo. The median refractive index was further decreased for the embryos in the later developmental stages. We note that the embryos used for the imaging were fixed with alcohol and mounted with Eukitt, which may have altered the refractive index values from their native values. Still, the change in the refractive index shown in Figure 8 suggests a condensation of the intracellular materials when the embryo progresses from the egg to the two-cell stage, then gradually decrease as the embryo develops further.

### 3.3. 3D Point Spread Function of the SWIR Digital Holographic Tomography System

**Figure 9:**
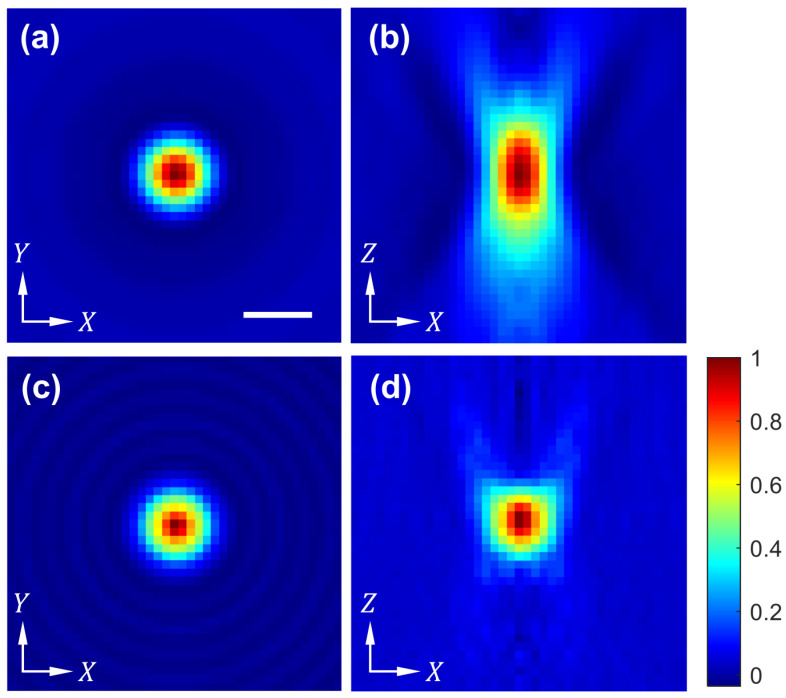
3D point spread function (PSF) of the developed instrument measured with gold nanoparticles (GNPs). (**a**,**b**) The horizontal and vertical cross-sections, respectively, of the 3D PSF before applying the regularization. (**c**,**d**) The horizontal and vertical cross-sections, respectively, of the 3D PSF after applying 30 iterations of the regularization. The 3D PSF measured with GNPs was averaged along the polar coordinate in each horizontal cross-section. The transverse and axial resolutions determined with the full-width at half maximum (FWHM) are 1.43 μm and 3.53 μm before the regularization, and 1.51 μm and 1.57 μm after the regularization. The scale bar (2 μm) in (**a**) applies to all the images.

We measured the 3D point spread function of the developed system using gold nanoparticles (GNPs) with a nominal diameter of 100 nm. In the raw interferogram images, we occasionally observed small dots of reduced intensity. Because the speckle noise could generate similar dots, we identified clusters of GNPs by looking for dots that remained stationary as we scanned the illumination angle. Gold has a very different refractive index from water or Eukitt. For example, the refractive index of gold is 0.733 at 1100 nm. Due to the large refractive index difference between gold and the mounting medium, the absorption measurement is highly affected by the scattering. For the same reason, the refractive index value obtained with DHT becomes hard to interpret. To address these challenges, we only used the refractive index profile of GNPs to assess the resolution. We also used the reconstruction algorithm based on the first-order Born approximation, which is more appropriate for small samples and less sensitive to refractive index mismatch [27]. From the reconstructed 3D map, we obtained the horizontal (XY) and vertical (XZ) cross-sections, including the pixel with the highest value, as shown in Figure 9a and Figure 9b, respectively. The full-width at half maximum (FWHM) values were used as measures of the transverse and axial resolution. The measured resolution was 1.43 μm (transverse) and 3.53 μm (axial) before applying the regularization. To image large samples, we sacrificed the spatial resolution. The current design of our DHT system is not diffraction limited, and the theoretical resolution is 1.44 μm, matching the measured transverse resolution. Figure 9c,d show the horizontal (XY) and vertical (XZ) cross-sections of the 3D refractive index map after applying 30 iterations of the regularization. The resolution was measured to be 1.51 μm (transverse) and 1.57 μm (axial). The axial resolution was significantly improved, while the transverse resolution was slightly lowered.

## 4. Discussion

Research on the optical properties of human hair has a wide range of practical applications in various fields, including cosmetic science [28], computer graphics [29], forensics [30], medicine [22], and anthropology [31]. In particular, the optical properties of hair in the SWIR range will allow for accurate modelling of light–skin interactions in deep-tissue imaging [32]. The information may also increase the accuracy of face recognition using the SWIR light, known to distinguish artificial hairs from natural ones [33].

The method demonstrated here could be useful for a wide range of microscopic specimens showing the absorption or refractive index contrast in the SWIR range. For example, the developed system could be used to detect microplastics (e.g., polystyrene, polypropylene, polyethylene), which show distinct absorption and refractive index spectra in the SWIR range [34]. FTIR and Raman are the dominant methods, but they only provide the type, not the quantity (i.e., volume), of the microplastic. In contrast, the DHT system can provide the quantity as well as the type of microplastic. Another potential application is to measure the optical properties of pharmaceutical powders in the SWIR range, providing an important input to the analysis of SWIR hyperspectral imaging used for the inline quality control of pharmaceutical products [35].

There are numerous research studies using DHT for live-cell imaging [36]. The refractive index measurement in the SWIR range has an advantage in imaging thick specimens, such as embryos. DHT in the visible range is typically applied to thin specimens, because the phase unwrapping often fails with thick specimens. This problem can be alleviated in the SWIR range, because the phase alteration is inversely proportional to the wavelength; thus, an abrupt phase jump near the boundary is reduced.

The anisotropic resolution due to the missing cone is a well-known problem with beam-rotation DHT. Although the regularization improves the axial resolution and accuracy of refractive index prediction, it should be used with caution, as the results depend on the type of constraints and various hyperparameters, such as the number of iterations [37]. Combining beam rotation with sample rotation can provide near-isotropic resolution [14,15], although the data acquisition time significantly increases.

## 5. Conclusions

We have demonstrated spectroscopic microtomography in the SWIR using digital holographic tomography and a wavelength-scanning laser. Applying the developed instrument to a human hair sample, we have measured the absorption coefficient and refractive index in the wavelength range of 1100–1650 nm at the 50 nm step size allowing for structural and chemical imaging without the need for special sample preparation. Further, we have measured the index of refraction of sea urchin embryos in six stages of development, showing the condensation and expansion of intracellular materials. Using GNPs, the 3D point spread function was measured and the resolution was improved from 1.43 μm (transverse) and 3.53 μm (axial) to 1.51 μm (transverse) and 1.57 μm (axial) by using regularization.

## Abbreviations

The following abbreviations are used in this manuscript:

MIR	Mid-infrared
NIR	Near-infrared
SWIR	Short-wave infrared
DHT	Digital holographic tomography
TBPF	Tunable bandpass filter
OPD	Optical path difference
VOPL	Variable optical path length
LED	Light-emitting diode
